# A Neural Correlate of the Processing of Multi-Second Time Intervals in Primate Prefrontal Cortex

**DOI:** 10.1371/journal.pone.0019168

**Published:** 2011-04-27

**Authors:** Naosugi Yumoto, Xiaofeng Lu, Thomas R. Henry, Shigehiro Miyachi, Atsushi Nambu, Tomoki Fukai, Masahiko Takada

**Affiliations:** 1 Department of System Neuroscience, Tokyo Metropolitan Institute for Neuroscience, Tokyo Metropolitan Organization for Medical Research, Fuchu, Tokyo, Japan; 2 Department of Neurology, School of Medicine, University of Minnesota, Minneapolis, Minnesota, United States of America; 3 Clinical Neuroscience Center, University of Minnesota Medical Center, Fairview, Minneapolis, Minnesota, United States of America; 4 Department of Neurophysiology, School of Medicine, Juntendo University, Tokyo, Japan; 5 Cognitive Neuroscience Section, Primate Research Institute, Kyoto University, Inuyama, Aichi, Japan; 6 Division of System Neurophysiology, National Institute for Physiological Sciences, Okazaki, Aichi, Japan; 7 Laboratory for Neural Circuit Theory, RIKEN Brain Science Institute, Wako, Saitama, Japan; 8 Systems Neuroscience Section, Primate Research Institute, Kyoto University, Inuyama, Aichi, Japan; Pennsylvania State University, United States of America

## Abstract

Several areas of the brain are known to participate in temporal processing. Neurons in the prefrontal cortex (PFC) are thought to contribute to perception of time intervals. However, it remains unclear whether the PFC itself can generate time intervals independently of external stimuli. Here we describe a group of PFC neurons in area 9 that became active when monkeys recognized a particular elapsed time within the range of 1–7 seconds. Another group of area 9 neurons became active only when subjects reproduced a specific interval without external cues. Both types of neurons were individually tuned to recognize or reproduce particular intervals. Moreover, the injection of muscimol, a GABA agonist, into this area bilaterally resulted in an increase in the error rate during time interval reproduction. These results suggest that area 9 may process multi-second intervals not only in perceptual recognition, but also in internal generation of time intervals.

## Introduction

**Figure 1:**
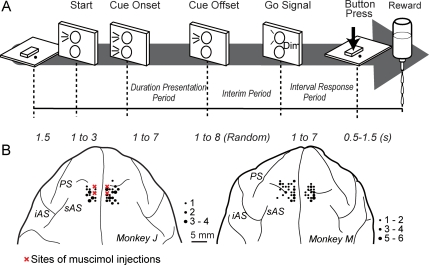
Task schema and recording sites. (A) Behavioral task schema. The monkeys were trained to prepare for and then observe the presentation of a time interval of visual stimuli, and after a variable interim period, then to reproduce this presented interval with a button press, as described in materials and methods. (B) Sites of single unit recordings and muscimol injections. Each dot indicates an electrode track where cellular activity was recorded in relation to the behavioral task. The size of the dot is proportional to the number of task-related cells in area 9. Red crosses denote sites of muscimol injection, which was performed to analyze effects on performance of the behavioral task. iAS, inferior limb of the arcuate sulcus; PS, principal sulcus; sAS, superior limb of the arcuate sulcus.

Time is a fundamental element in living systems [1]. When we speak, or play sports and music, we sense the elapsed time intervals to monitor the events, and even generate preferred durations for the completion of the performance of the task. Other species also rely on perception of time to coordinate their behavior [1]–[3]. Brain mechanisms for tracking temporal features of external stimuli are known to utilize neuronal assemblies of the cerebellum [4], [5], olivo-cerebellar system [6], [7], basal ganglia [8], cortico-striatal circuits [9]–[13] and cerebral cortex [14]–[19]. Subcortical areas, particularly within the olivo-cerebellar system, can process measures of time for motor control on the order of milliseconds [6]. Cortical areas, particularly frontal or prefrontal cortex (PFC), may be involved in cognitive tasks such as time estimation [20], time discrimination [21], frequency timing [22], and timing of delay [23]. Recognition of multi-second intervals of external stimuli may require processing in PFC [24]. However, it remains unclear whether the PFC is involved in generation of multi-second time intervals, without reference to environmental stimuli. To address this question, we devised a time-reproduction task similar to tasks studied in human subjects [25], which required two macaque monkeys to estimate specific multi-second time intervals during stimuli (durations of 2, 4, and 7 s for monkey J, and 1 and 5 s for monkey M), and then later to reproduce these intervals by pressing a button based on an internally generated estimate of the elapsed time (Fig. 1 A). The principal features of our task were as follows: (1) The target duration was presented for a specific multi-second interval (from among a set of intervals for which the monkey had been trained); (2) The monkey needed to perceive the time elapsed during this presentation period, in order to reproduce the interval later; (3) After a variable interim period, the monkey had to actually reproduce the time interval that matched the interval previously presented, in order to receive the reward. Thus, this task enabled us to investigate the neuronal activity associated with both perception and reproduction of time by means of extracellular single unit recording in area 9 of the PFC during performance of the task. In addition to the extracellular single unit recording in area 9, we performed muscimol blockage in area 9 to investigate whether reversible ablation of this site would induce behavioral changes on comparing pre-versus post-injection data.

## Methods

### Animals

We used two macaque monkeys (Macaca fuscata): monkey J (6.1 kg) and monkey M (5.6 kg). This study was carried out in strict accordance with the Guideline for the Care and Use of Animals (Tokyo Metropolitan Institute for Neuroscience 2000). All surgical and experimental protocols were approved by the Animal Care and Use Committee of the Tokyo Metropolitan Institute for Neuroscience (Permit Number:08–1815). All efforts were made to minimize suffering in accordance with the recommendations of the “The use of non-human primates in research”. For example, the monkeys were kept in individual primate cages in an air-conditioned room where food was always available. Their health condition, including factors such as body weight and appetite, was checked daily. Supplementary water and fruit were provided daily. All surgery was performed under general anesthesia (intravenous injection of pentobarbital sodium).

### Behavioral procedures

The time-reproduction task required the monkey to estimate specific multi-second durations during signal presentations, and then to reproduce these durations by planning the interval response (button press) based on estimates of the elapsed times. During each stimulus-response trial, the time task began with moving a hand to a light sensor, a black dot beside button, and continuously leaving the hand on the sensor for 1.5 s (Fig. 1A). A control LED on a vertical plate fixed directly in front of the monkey was turned on. After 1–3 s, another LED (instruction LED) was turned on and lasted 2, 4, or 7 s for monkey J and 1 or 5 s for monkey M, to signal the time intervals that they had to reproduce later. Following an additional interim period (randomly assigned as 1–8 s), the control LED dimmed (Go signal). On observing a dimming of the LED (the “Go signal”, to signal the start of the interval response period), the monkey had to reproduce the time interval that matched the interval previously presented; then the monkey pressed a button to signal the end of the interval response period (reproduced intervals) (Fig. 1 A). Successful trials were defined as intervals reproduced within±15% of the interval previously presented, which was defined as the “correct response range (CRR)”. The successful trials were always followed by supply of liquid reward.

### Surgical and electrophysiological recording procedures

The monkeys were trained to perform the task consistently with greater than 80% accuracy (i.e., with 80% of responses of generated intervals that fell within the CRR). At the final stage of the training period, a head holder and a chamber for unit recordings were implanted. The surgical and electrophysiological recording procedures were described in detail elsewhere [26], [27]. We performed single unit recordings using a glass-coated Elgiloy-alloy microelectrode (0.5–1.5 MOhm at 1 kHz). During the recording, the time was chosen from a set either of 2, 4, and 7 s, or of 1 and 5 s. In order to prevent habituation to the performance of specific times, times were presented pseudo-randomly for each repetition, at least five repetitions for each cell. Eye and hand movements were monitored by a video camera while the monkey's head was fixed to the primate chair.

We identified the sites of single unit recordings primarily as area 9 according to the following procedures: (1) pre-operative MRI images (Hitachi, AIRIS, 0.3 T) to determine the best position of a recording chamber [26]; (2) anatomical location (dorso-medial) PFC, 1–6 mm from midline, anterior to the near end of the superior arcuate sulcus; (3) cortical surface reconstruction of electrode penetrations in the post-mortem brains (see Fig. 1B).

### Muscimol injections

We used a stainless-steel tube (inner diameter 0.06 mm, outer diameter 0.14 mm, length 180 mm) with a sharp angle at the tip, to which a tungsten microelectrode (impedance 0.5–2.0 MOhm at 1 kHz) was attached side by side with an instant glue, where the tip of the electrode protruded from the tip of the injection tube by 0.2–0.3 mm. The injection tube was connected to a 10-µl Hamilton microsyringe by a polyethylene tube (diameter, 0.3 mm). We carried out a total of three muscimol injection experiments in monkey J, each on a separate day in order to make reversible inactivation of the PFC. During an injection experiment, we first recorded neuronal activities using the microelectrode attached to the injection tube. Injections were made at the depth that the task-related neurons were observed. The injections were always done into both hemispheres of the brain, two sites on each hemisphere (Fig. 1B). An aqueous solution of muscimol (Sigma; 5 µg/µl) was pressure-injected in 5–7 steps (0.2 µl for each step) with an interval of 20 s between steps. A total amount of 1.0–1.4 µl was deposited for each injection site. We collected behavioral data for 3 hours after the injections.

We chose not to perform saline control injections at this site, given evidence that there was no effect after a similar amount of saline was injected into multiple areas of the primate brain, such as cortex [28], or cerebellar dentate nuclei through the same procedure [26], we did not perform saline injections for the current study.

### Data analysis

To define “duration-recognizing” (DR) neurons and “interval-generating” (IG) neurons, we first examined whether discharge rates during the interim period and the interval-response period significantly varied among different presented intervals (2 s, 4 s, and 7 s for monkey J; 1 s and 5 s for monkey M; ANOVA, P<0.05). Second, if the discharge rate for a certain interval (e.g. 2 s) was significantly higher than those for the others (4 s or 7 s) (Fisher's SLD test, P<0.05) during the interim period, the neuron was defined as the DR neuron, specific for the interval (e.g., DR neuron, 2-s specific neuron). If the discharge rate for a certain interval (e.g. 2 s) was significantly higher than those for the others (4 s or 7 s) (Fisher's SLD test, P<0.05) during the interval-response period, the neuron was defined as the IG neuron, specific for the interval (e.g., IG neuron, 2-s specific neuron).

We compared the error rate of the post-injection performance with that of the pre-injection performance to assess the effect of muscimol blockade of prefrontal cell activity on the monkey's performance. The error rate was calculated as the ratio of failed trials to the total of failed and successful trials during the performance of a block of 10 successful repetitions. Pre-injection data and post-injection data were collected in 3 paired days separated by one week between pairs, with a pre-injection session on one day and a muscimol injection session on the following day. Statistical comparison (t-test, P<0.05) was made for the error rates between the pre- and post-muscimol injections in the three injection experiments. A total of 1080 and 1134 trials of task performance, approximately 360 and 378 trials per time interval, were included in the post- and pre-injection groups, respectively. A button press frequency (a response rate) was calculated as the ratio of the number of responses during 50 ms time bin to the total of 360 or 378 trials.

## Results

### Activity during duration recognition

We found two groups of time related neurons, with single unit recordings carried out in area 9 of the PFC during performance of the time task. One group showed a higher activity lasting 1–2 s immediately after the duration-presentation period, with specificity of individual neurons to particular intervals (Fisher's SLD test, P<0.05). We termed such neurons “duration-recognizing” (DR) neurons. Another group showed increased activity during the interval response period (time-reproduction period), with specificity of individual neurons to particular intervals (Fisher's SLD test, P<0.05). We termed these neurons “interval-generating” (IG) neurons. Among 497 cells (154 cells in monkey J; 343 cells in monkey M) recorded from the PFC, the DR cells constituted 39% (n = 60) in monkey J and 29% (n = 98) in monkey M, and the IG cells constituted 44% (n = 68) in monkey J and 32% (n = 111) in monkey M. Only a small group of neurons, 9% (n = 14) in monkey J and 3% (n = 10) in monkey M were active during both the interim and interval response periods. This indicates that DR and IG functions were rarely combined in a single cell.

**Figure 2:**
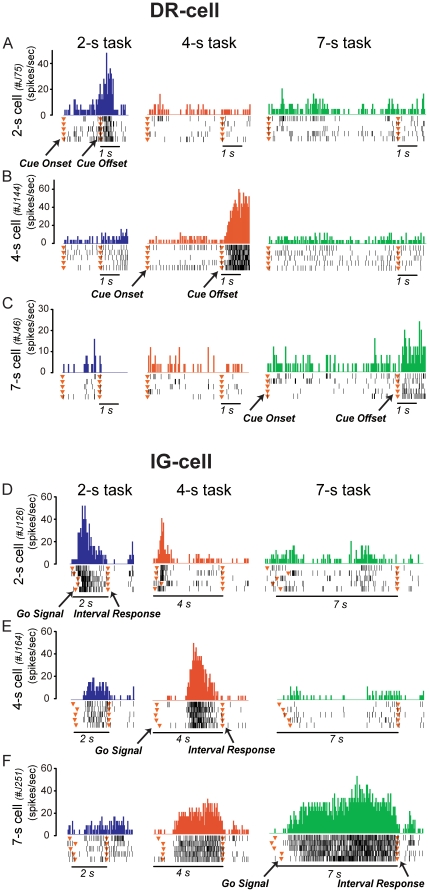
Duration-recognizing- and interval-generating-related activity. Activity of individual DR neurons specific for 2 s (A), 4 s (B), or 7 s (C) in monkey J. Shown in histogram and raster format is spike discharge during the duration presentation period and the early interim period of each time task. Note the time-specific activity that is seen during the 1-s period after cue offset. Activity of individual IG neurons specific for 2 s (D), 4 s (E), or 7 s (F) in monkey J. Shown is the spike discharge rate during the interval response period of each time task.

Typical activities of DR neurons in monkey J are shown in Fig. 2A–C, with examples of one neuron tuned to each of the time intervals (2, 4 and 7 s). Typical activities of DR neurons in monkey M, in cells specific for 1 and 5 s, are depicted in Fig. S1. This is most evident if one compares neuronal discharges during the initial 1-s portion of the interim period across the different time intervals. The cell in Fig. 2A showed higher activity after 2-s interval presentation than after 4-s and 7-s interval presentations (Fisher's SLD test, P<0.05). Similarly, the cells in Fig. 2B and 2C were tuned to 4-s and 7-s intervals, respectively (Fisher's SLD test, P<0.05). We propose that such time-specific activity may contribute to recognition of particular multi-second time lengths in environmental stimuli.

### Activity during time interval generation

The IG neurons shown in Fig. 2D–F demonstrated activities specific for 2, 4, and 7 s by increased firing during the interval-response period (Fisher's SLD test, P<0.05). Typical activities of IG neurons in monkey M, in cells specific for 1 and 5 s, are depicted in Fig. S2. For example, the cell (J164) in Fig. 2E showed more activity during the reproduction of the 4-s time length than it did during the 2-s and 7-s reproductions (Fisher's SLD test, P<0.05). Likewise, the cells J126 and J251 in Fig. 2D and 2F were more active during the reproduction of either the 2-s or the 7-s time period, respectively, than they were during other interval reproductions. We propose that this type of time-specific activity is involved in generating an internal representation of time length that is at least partly independent of external stimuli.

Each monkey had approximately equal proportions of DR neurons and IG neurons tuned to each of the highly practiced time intervals. Among 60 DR cells in monkey J, 38% (n = 23), 27% (n = 16), and 35% (n = 21) of the total exhibited activities specific for presented durations of 2, 4, and 7 s, respectively. Among 68 IG cells in this monkey, 40% (n = 27), 28% (n = 19), and 32% (n = 22) of the total showed 2-s, 4-s, and 7-s specific activities, respectively. Among 98 DR cells in monkey M, 58% (n = 57) and 42% (n = 41) of the total were tuned to 1-s and 5-s durations, respectively. Among 111 IG cells in this monkey J, 50% (n = 56) and 50% (n = 55) of the total were tuned to 1-s and 5-s durations, respectively.

On the other hand, only a small group of neurons were more active during the duration-presentation period. In monkey J, 3% (n = 5) and in monkey M 6% (n = 20) of the total of recorded cells had enhanced activity early during presentation of the time intervals (ANOVA, p<0.05). We failed to detect significant relationships between the firing patterns of these neurons and the specific time intervals.

**Figure 3:**
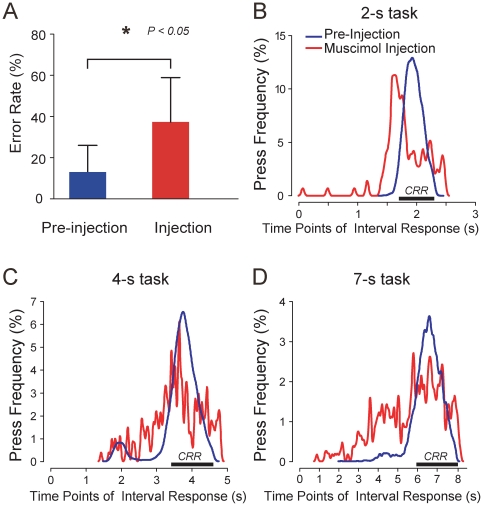
Effect of muscimol injections into area 9 on the accuracy of time reproduction in monkey J. (A) Change in the error rate for all of the 2-s, 4-s, and 7-s tasks. (B–D) Comparison of the frequency of interval responses between the pre- and the post-injections in the 2-s (B), 4-s (C), and 7-s (D) tasks. CRR, correct response range (reproduction accuracy within±15% of the target interval).

To further test the importance of area 9 neurons in the reproduction of time intervals, we reversibly inactivated the PFC in monkey J, by local injection of muscimol, a GABA agonist [26], [28]. The effect of muscimol on the accuracy of interval responses was demonstrated by a significant increase in the error rate for all three injections (Fig. 3A, t-test, P<0.05). Fig. 3B–D showed the further details of the behavioral changes with the comparison of the frequency of interval responses based on the estimates of the elapsed times between pre- and post-injection. The response times in the absence of muscimol injection were distributed with single peaks that fell nearly at the mid-point of the CRR and with relatively tight clustering around the CRR, but after muscimol injection the response times were more widely distributed and most errors occurred as excessive shortening of the response times (see Fig. 3B–D). It was noteworthy that a peak of the interval response density tended to shift earlier (Fig. 3B–D). The tendency toward excessively early button presses indicated that interference specifically with hand movements was unlikely to be the cause of inaccurate interval signaling. Thus, the PFC inactivation data provided additional evidence for the role of area 9 neurons in time reproduction.

## Discussion

Our data demonstrate that time is represented in the PFC or neural networks involving the PFC. Previous studies have shown that neurons in the PFC participate in many aspects of cognitive behaviors based on reward [29], evaluating self-generated decisions [30], categorization [31], procedural learning [32], functional separation of “what” and “when” [33], and time prediction and detection [34]. These earlier observations encouraged our detailed analysis of area 9 neuronal activities in critical aspects of temporal processing.

An important finding in our study was that a group of PFC neurons (DR neurons) displayed activities just after the presentation of the target duration ended, which were specific for multi-second intervals presented during the duration-presentation period. Time-related neuronal activity has been reported in various motor areas of the primate frontal cortex, such as the dorsal premotor cortex [35], the presupplementary motor area (pre-SMA) [36], [37] and the supplementary eye field (SEF) (23). Repetitive transcranial magnetic stimulation shows the evidence of role of the dorsolateral prefrontal cortex in short (0.5 s) and long (2 s) interval timing in human subjects [38]. In a rather different task not involving the reproduction of time intervals, Genovesio et al. have shown that there was post-delay spike activity in areas 46, 8, 9, and rostral 6 that was specific for each of the elapsed delay periods (1 s, 1.5 s, and 2 s) in primates [19]. Yet, Matell, Meck, Jin, and their colleagues have provided strong evidence of neural representation of multi-hundred millisecond time in dorsolateral PFC-basal ganglia circuits [39], [40], [41]. From these observations, a hypothesis arises that PFC neurons or the related neural networks may change their activities by practice in response to varying elapsed times, thereby detecting or recognizing individual time lengths up to 7 seconds.

Beyond the time-perceptive neurons, the present study has revealed that, during the interval-response period, another group of PFC neurons (IG neurons) displays higher activity specific for different presented time lengths. Our results have clearly demonstrated that, in the primate, there are PFC neurons that can generate distinct time intervals up to 7 seconds. This may provide a useful clue for understanding how signals derived from DR neurons are decoded to motor output, in order to control the timing of the button press after the time interval. We hypothesize that these IG neurons may provide this control.

Given the theory that striatal activity may be the final output of an internal clock [10], and the anatomical evidence that the striatum receives input from area 9 [42], the cortico-striatal projection from area 9 may play a key role in the temporal command for action. Others have suggested that corticostriatal interactions may be critical to reward-enhanced learning [43], and future studies might address how area 9 neurons become tuned to specific multisecond time intervals by simultaneously recording area 9 and striatal neurons during training for such tasks.

Is it possible that the time interval-specific activity that we documented was merely an epiphenomenon? We think not, for several reasons. First, the time interval-specific activity was highly represented among cortical cells in the area 9. The cells involved in time interval, either the DR cells or the IG cells were not a small subpopulation, but approximately formed one out of three of the whole population under study. This proportion of time interval cells in cortical area 9 was similarly observed between two monkeys in the current study. Further, for each of the highly practiced time intervals, each monkey had approximately equal proportions of the DR neurons and of IG neurons, while it was rare that DR and IG functions were combined in a single cell. Second, our recording location, area 9 is characterized by a particular firing pattern of the full layer cortex construction that is distinguishable from the posterior motor areas, which lack layer IV. Accordingly, we did not find evidence that area 9 cells responded to eye movements or hand movements which occurred during the responses used to indicate the internally generated time intervals. The task in our study required only limited eye and hand movements. The monkeys placed the hand on a sensor point at the beginning of the trial, and kept the hand on that point until the end of the trial, after reward delivery. To indicate the internally generated time interval, the monkey needed to move the thumb only a few millimeters to press the button. We monitored eye movements and hand movements, but we did not see individual area 9 neurons that responded to eye or hand movements that occurred during our task. These observations indicated that our recording area was separated from motor areas such as the pre-SMA or SEF. Finally, the most direct evidence of the involvement of prefrontal cortex comes from the results of muscimol interference. We found that the accuracy of time interval production was disrupted.

In conclusion, different groups of PFC neurons in area 9 had enhancement in neuronal discharge just after the duration-presentation period or during the interval-reproduction period, with tuning to specific lengths of time. These results suggest that the PFC neurons contribute to both perception and generation of multi-second time intervals.

## Supporting Information

Figure S1 **Duration-recognizing-related activity.** Activity of individual DR cells specific for 1 s (A) or 5 s (B) in monkey M. Shown in histogram and raster format is spike discharge during the interim (post-duration-presentation) period of each time task. Note the time-specific cell activity that is seen during the 1-s period after cue offset.
(TIF)

Figure S2 **Interval-generating-related activity.** Activity of individual IG cells specific for 1 s (A) or 5 s (B) in monkey M. Shown in histogram and raster format is spike discharge during the interval-response period of each time task.
(TIF)
