# Supplementary figures and images for: A Neural Correlate of the Processing of Multi-Second Time Intervals in Primate Prefrontal Cortex
Source: PLoS One. 2011 Apr 27;6(4):e19168. doi: 10.1371/journal.pone.0019168 (PMC3083430; doi:10.1371/journal.pone.0019168)

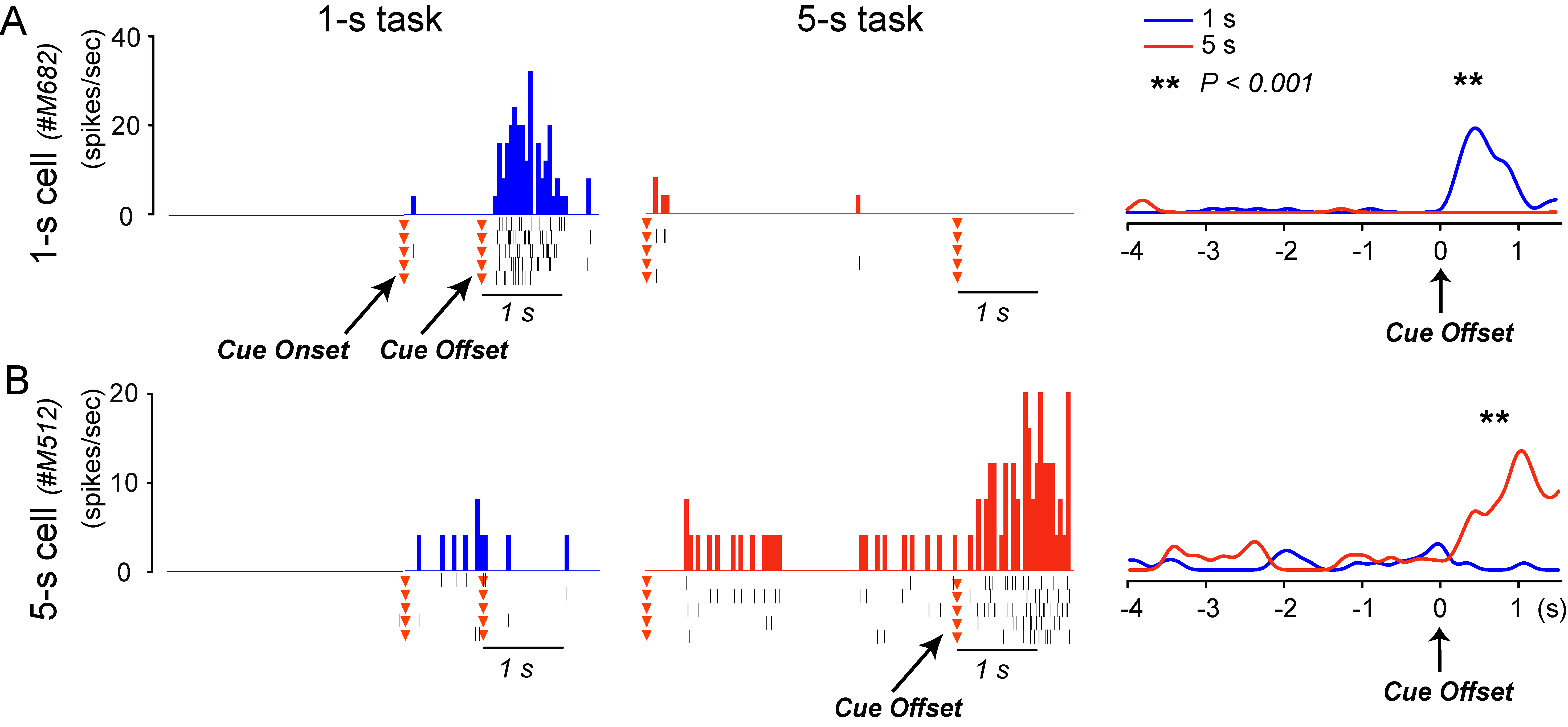

Supplement: Figure S1 — Duration-recognizing-related activity. Activity of individual DR cells specific for 1 s (A) or 5 s (B) in monkey M. Shown in histogram and raster format is spike discharge during the interim (post-duration-presentation) period of each time task. Note the time-specific cell activity that is seen during the 1-s period after cue offset. (TIF) [file pone.0019168.s001.tif]

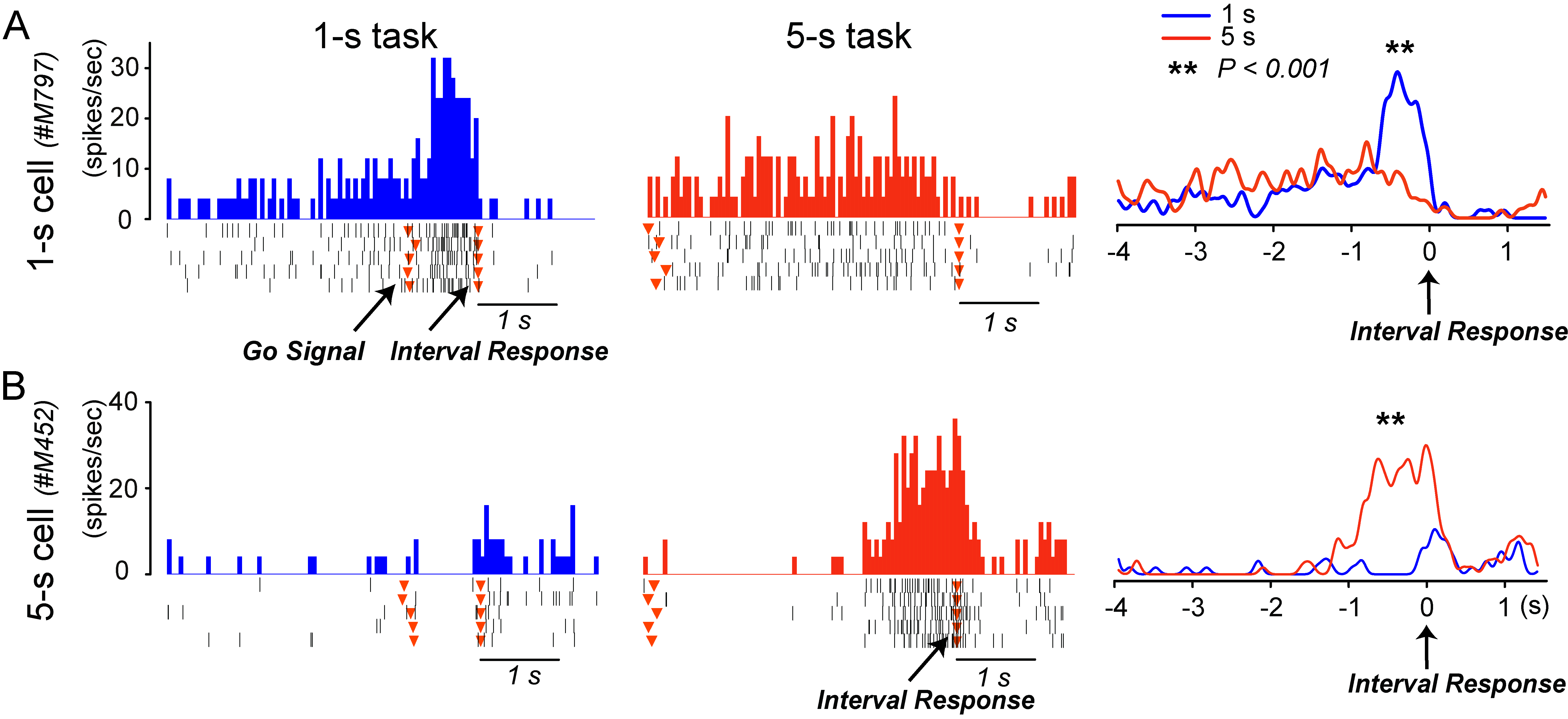

Supplement: Figure S2 — Interval-generating-related activity. Activity of individual IG cells specific for 1 s (A) or 5 s (B) in monkey M. Shown in histogram and raster format is spike discharge during the interval-response period of each time task. (TIF) [file pone.0019168.s002.tif]
